# Retrospective Serologic Assessment of Vitamin D Levels in Children from Western Romania: A Cross-Sectional Study

**DOI:** 10.3390/medicina61030394

**Published:** 2025-02-24

**Authors:** Alin Gabriel Mihu, Cristian Mircea Nicolescu, Constantin Catalin Marc, Casiana Boru, Monica Susan, Alina Ciceu, Sergiu Adrian Sprintar, Alexander Tudor Olariu, Daniela Adriana Oatis, Laura Corina Nicolescu, Tudor Rares Olariu

**Affiliations:** 1 Center for Diagnosis and Study of Parasitic Diseases, Department of Infectious Disease, Victor Babes University of Medicine and Pharmacy, 300041 Timisoara, Romania; alin.mihu@umft.ro (A.G.M.); catalin.marc@umft.ro (C.C.M.); sergiu.sprintar@umft.ro (S.A.S.); rolariu@umft.ro (T.R.O.); 2 Bioclinica Medical Analysis Laboratory, Dreptatii Street, No. 23, 310300 Arad, Romania; 3 Department of Biology and Life Sciences, Vasile Goldis Western University of Medicine and Pharmacy, 310025 Arad, Romania; cristian_ans@yahoo.com; 4 Department of Medicine, Vasile Goldis Western University of Medicine and Pharmacy, 310025 Arad, Romania; boru.casiana@uvvg.ro; 5 Centre for Preventive Medicine, Department of Internal Medicine, Victor Babes University of Medicine and Pharmacy, Eftimie Murgu Square, No. 2, 300041 Timisoara, Romania; susan.monica@umft.ro; 6 Aurel Ardelean Institute of Life Sciences, Vasile Goldis Western University of Arad, 310414 Arad, Romania; ciceu.alina@uvvg.ro; 7 Patogen Preventia, 300124 Timisoara, Romania; alexanderolariu@yahoo.com; 8 Discipline of Parasitology, Department of Infectious Diseases, Victor Babes University of Medicine and Pharmacy, 300041 Timisoara, Romania; 9 Clinical Laboratory, Municipal Clinical Emergency Teaching Hospital, 300254 Timisoara, Romania

**Keywords:** 25-hydroxyvitamin D, seasonal variation, epidemiological research, Romania

## Abstract

*Background and Objectives:* Adequate levels of vitamin D are vital for both growth and immunomodulation in children. To evaluate the levels of vitamin D in children from Western Romania and to identify significant age, seasonal, and geographical disparities. *Materials and Methods:* This study evaluates the level of 25-hydroxyvitamin D levels assessed on Cobas 6000’s module e601 in 1698 children aged 1–18 years between 1 January 2018 and 31 December 2021 from Western Romania. *Results*: Children aged 1–6 years predominantly present sufficient levels (>30 ng/mL), while older age groups showed a marked decline. Adolescents aged 13–18 years were most affected, with over half displaying insufficient levels (20–30 ng/mL). Rural children were more likely to achieve sufficiency compared to urban peers. Males demonstrated significantly higher vitamin D levels when compared to females. Seasonal variations showed the highest vitamin D levels during late summer and early autumn (September: aOR = 5.47; 95% CI: 3.17–9.42, *p* < 0.001). Multivariate analysis revealed a significant improvement in vitamin D levels during 2019–2020. *Conclusions*: Our findings suggest the need for targeted screening programs and health policies to address vitamin D deficiency, particularly among older children, urban residents and during winter months.

## 1. Introduction

Vitamin D is a fat-soluble vitamin, synthesized in the skin during sun exposure in humans [1,2]. It plays an important role in bone growth and remodelling by stimulating the proper mineralization of bones and bone mass, being essential for maintaining calcium, phosphate, and magnesium homeostasis [3,4]. Vitamin D status is a determining factor for growth, influencing body composition and metabolic health [5]. Furthermore, vitamin D affects somatic growth and macronutrient metabolism by regulating the cell cycle and cell proliferation [4].

Vitamin D was reported to function as an immunomodulatory hormone [6], with a significant role in strengthening the immune system [7]. Through its actions on the macrophage function, dendritic cells, and T cells, vitamin D is a mediator of both innate and adaptive immunity [8]. The active form of vitamin D, namely 1,25-dihydroxyvitamin D, has been shown to have a significant influence on various components of the innate and adaptive immune system, as well as on endothelial membrane stability [6].

Vitamin D can be synthesized endogenously following occasional exposure of the skin to ultraviolet B radiation (290–350 nm), which facilitates the conversion of the 7-dehydrocholesterol molecule, present in the epidermis, into pre-vitamin D. It can also be obtained from dietary sources or supplements [9,10,11,12,13,14]. Around 5–10% of the vitamin D in the body comes from dietary intake, while over 90% is synthesized through skin exposure to sunlight [15]. The synthesis of vitamin D induced by sun exposure was reported to be influenced by factors such as season, time of day, duration of sun exposure, latitude, altitude, air pollution, skin pigmentation, use of sunscreen and the aging process [16,17].

Maintaining an optimal level of vitamin D is essential during the growth period for bone mass formation in childhood and, subsequently, for the prevention of osteoporosis in adulthood [18,19,20]. Children are more likely to exhibit vitamin D deficiency, which negatively affects mineral metabolism, causing growth retardation and bone deformities [21]. Considering that bone mass is accumulated during childhood and adolescence and peak bone mass is reached [21], optimizing bone mineral density during childhood and adolescence plays an important role in maintaining skeletal health [1]. Evaluating vitamin D status is particularly important during childhood because growth in height and active bone formation may involve needs that differ from those of the adult population [22].

In developed nations such as the USA, Great Britain, and Turkey, studies found a considerable proportion of children presented insufficient or deficient vitamin D levels, with 9% of the children from the USA being deficient and 40% of Turkish children falling below the deficiency threshold of 20 ng/mL [23,24,25]. In contrast, developing countries often reported even higher vitamin D deficiency rates compared to developed countries. Studies indicate that in India and Nepal, over 80% and 91% of children, respectively, were deficient in vitamin D [26,27]. Similarly, a report from Sub-Saharan Africa found a prevalence of vitamin D deficiency of around 50% among healthy children [28].

Currently, limited data regarding the prevalence of vitamin D deficiency and insufficiency are available in Romania [29,30,31,32] and no studies have been conducted recently in Western Romania. Due to this reason, we decided to assess the levels of vitamin D in children from Western Romania and the monthly/yearly timing.

## 2. Materials and Methods

### 2.1. Study Design, Population and Data Collection

Our study was designed as a cross-sectional, retrospective study. We included 1698 consecutive children who came in for a routine blood draw at Bioclinica Clinical Laboratories, between 1 January 2018 and 31 December 2021. Bioclinica Clinical Laboratories has several collection points in both rural and urban areas of Arad County. In order to ensure full participant anonymity, the dataset included demographic information for each participant such as the age group at the moment of the blood draw (1–6 years, 7–12 years and 13–18 years), area of residence (rural or urban), gender assigned at birth (female or male), year and month of the blood draw as well as vitamin D status (severely deficient, deficient, insufficient, sufficient).

### 2.2. Inclusion and Exclusion Criteria

We included consecutive, apparently healthy, children who came for a routine blood draw who were residents of Arad County and their primary caregiver allowed participation.

Children whose primary caregiver did not consent to participate were excluded. Also, children with severe diseases such as severe metabolic disorders or cancers, children taking medications that could affect vitamin D levels (corticosteroids, anticonvulsants) and children presenting anorexia or obesity were excluded from the study.

### 2.3. Sample Collection and Laboratory Assessments

From all the study participants, venous blood samples into serum clot activator tubes were collected using the standard venipuncture techniques between 7 a.m. and 11 a.m. All participants were in a fasted state, due to the overnight fasting. The filled clot activator tube was then centrifuged at 2000× *g* for 10 min, within an hour of collection, and then placed into Cobas 6000’s, module e601 (Roche Diagnostics, Mannheim, Germany) to assess the level of 25-hydroxyvitamin D, using electrochemiluminescence.

Levels of vitamin D above 30 ng/mL were considered sufficient, levels between 20 and 30 ng/mL were considered insufficient, levels between 12 and 20 ng/mL were considered to be indicative of deficiency and levels below 12 ng/mL were considered to representative of severe deficiency. All determinations conducted in this study were in accordance with the internal laboratory standards and with the manufacturer’s instructions.

### 2.4. Data and Statistical Analysis

Data were presented as numbers, percentages as well as mean ± standard deviation (SD). We used descriptive statistics to summarize the key characteristics of the study population. For continuous variables mean and standard deviation were used. Percentages were used for categorical variables.

All statistical analyses were performed with Stata 16.1 (StataCorp, College Station, TX, USA). Ordinal univariate logistic regression was chosen as the primary statistical model for this study, due to its appropriateness in analysing ordinal outcome variables. This model facilitated the exploration of associations between vitamin D levels (categorized as severely deficient, deficient, insufficient and sufficient) and various independent variables (age group, gender, area of residence, month and year of the blood collection). After identifying variables that reached statistical significance in the univariate model (*p* < 0.05), further investigation was conducted to determine if they remained relevant in a multivariate logistic regression analysis. We presented crude odds ratios (OR) with their corresponding 95% confidence intervals (95% CI) for each ordinal univariate logistic regression conducted. For the multivariate logistic regression model, adjusted odds ratios (aOR) along with their 95% CI were presented. For both univariate and multivariate logistic regression models, a *p*-value of <0.05 was considered to present statistical significance.

### 2.5. Ethical Approval

Ethical approval was obtained from Vasile Goldis University Ethics Committee, Arad, Romania (no. 15 from 31 March 2023).

## 3. Results

Assessment of vitamin D levels in 1698 children aged between 1 and 18 years from Western Romania, revealed that 32 (1.88%) had severe deficiency (<12 ng/mL), 247 (14.55%) had deficiency (12–20 ng/mL), 581 (34.22%) had insufficiency (20–30 ng/mL), and 838 (49.35%) had sufficient levels (>30 ng/mL). Of the 1698 children, 1309 were from urban regions and 886 were males (Table 1).

Children aged 1–6 years predominantly presented sufficient vitamin D levels (>30 ng/mL) and served as the reference group in the ordinal univariate logistic regression model. Children aged 7–12 years displayed a significantly lower likelihood (cOR = 0.17; 95% CI: 0.13–0.21, *p* < 0.001) of being in a higher category of vitamin D status compared to the reference group, with the majority having levels indicative of insufficiency (20–30 ng/mL). Adolescents aged 13–18 years presented even lower odds (cOR = 0.08; 95% CI: 0.06–0.11, *p* < 0.001) of being in a higher category of vitamin D status, with over half falling into the insufficiency range, indicating a significant deficiency as they aged.

Children from rural areas were more likely to be in a higher category of vitamin D status, with over half exceeding the sufficiency threshold of 30 ng/mL and were used as the reference group. Urban residents, in contrast, had significantly lower odds (cOR = 0.78; 95% CI: 0.63–0.97, *p* = 0.03) of exceeding the sufficiency (>30 ng/mL) range, with a smaller proportion in the highest category.

When females were used as the reference group, males showed a higher likelihood (cOR = 1.23; 95% CI: 1.02–1.47, *p* = 0.03) of achieving higher levels of vitamin D.

January was used as the reference month for the ordinal univariate logistic regression model, with 40.14% of children in the sufficient vitamin D category. Statistically significant differences were observed in July (cOR = 2.22; 95% CI: 1.41–3.49, *p* = 0.001), August (cOR = 3.21; 95% CI: 1.97–5.23, *p* < 0.001), September (cOR = 3.61; 95% CI: 2.22–5.88, *p* < 0.001), October (cOR = 2.87; 95% CI: 1.79–4.59, *p* < 0.001), and December (cOR = 1.88; 95% CI: 1.17–3.03, *p* = 0.01). No differences were observed in February (cOR = 1.09; 95% CI: 0.72–1.65, *p* = 0.67), March (cOR = 0.69; 95% CI: 0.46–1.04, *p* = 0.08), April (cOR = 0.95; 95% CI: 0.61–1.47, *p* = 0.81), May (cOR = 1.21; 95% CI: 0.80–1.82, *p* = 0.36), June (cOR = 1.47; 95% CI: 0.96–2.26, *p* = 0.08), and November (cOR = 1.5; 95% CI: 0.96–2.33, *p* = 0.08) (Table 2).

The year 2018 was the baseline year with most of the population having vitamin D levels in the sufficiency range (>30 ng/mL) and was used as a reference in the ordinal univariate logistic regression model. In 2019, a slight increase in the proportion of children with vitamin D levels in the sufficiency range (cOR = 1.19; 95% CI: 0.92–1.52, *p* = 0.19) was observed, although this change was not statistically significant. In 2020, the likelihood of children being in a higher category of vitamin D status, with an odds ratio of 1.33 (95% CI: 1.03–1.72, *p* = 0.03), was statistically significant compared to 2018. In 2021, a decrease in the proportion of individuals with sufficient vitamin D levels was observed, corresponding to a lower odds ratio (cOR = 0.81; 95% CI: 0.61–1.07, *p* = 0.13), though this decrease was not significant (Table 3).

Based on the multivariate logistic regression analysis, children aged 7–12 years presented significantly lower odds (aOR = 0.16; 95% CI: 0.13–0.21, *p* < 0.001) of being in the higher categories of vitamin D levels compared to the youngest age group, underscoring a decrease with age. Adolescents aged 13–18 years showed even lower odds (aOR = 0.07; 95% CI: 0.05–0.09, *p* < 0.001), suggesting a continued decline in vitamin D levels with further aging. The year 2019 exhibited a significant increase in the likelihood of higher vitamin D levels compared to 2018 (aOR = 1.49; 95% CI: 1.14–1.97; *p* = 0.004). The year 2020 marked the highest rise (aOR = 1.73; 95% CI: 1.29–2.32, *p* < 0.001), indicating the most substantial improvement in the odds of children being in a higher category of vitamin D status in the observed period. In 2021, the upward trend continued with a significant likelihood of improved vitamin D levels (aOR = 1.63; 95% CI: 1.19–2.24; *p* = 0.003). July showed a significant increase in the odds of a higher vitamin D category (aOR = 2.61; 95% CI: 1.58–4.30; *p* < 0.001). August further elevated the likelihood of higher vitamin D levels (aOR = 3.71; 95% CI: 2.16–6.38; *p* < 0.001). September demonstrated the peak odds (aOR = 5.47; 95% CI: 3.17–9.42; *p* < 0.001), highlighting the most substantial effect of seasonal sun exposure. October still showed significantly high odds (aOR = 4.52; 95% CI: 2.68–7.64; *p* < 0.001). November presented a decrease yet maintained significant improvement in vitamin D levels (aOR = 1.83; 95% CI: 1.13–2.96; *p* = 0.013). December ended the year with notable, albeit lower, odds compared to peak summer months (aOR = 2.2; 95% CI: 1.31–3.71; *p* = 0.003) (Table 4).

## 4. Discussion

This is the first study to assess vitamin D levels in non-hospitalized children from Western Romania for more than 10 years [30]. Our results revealed a 34.22% prevalence of vitamin D insufficiency among the pediatric population. Similar findings were reported in another study conducted in Southeastern Romania, between September 2021 and September 2023, where the prevalence of vitamin D insufficiency was 31.8% in hospitalized children aged 2–17 years [32]. Another study, conducted in Northeastern Romania by Ghiga et al. [31] between August 2019 and January 2024, assessed the 25-hidroxyvitamin D levels in hospitalized children and reported the following: 77/744 (10.34%) were severely deficient (<10 ng/mL), 221/744 (29.7%) were deficient (10–20 ng/mL), and in 194 (26.07%) insufficiency (20–29 ng/mL) was noted.

The prevalence of vitamin D deficiency was 14.55% among the participants in this study, and severe deficiency was observed in 1.88% of the children included in the study. In this study, vitamin D was lower compared to the results obtained by Badiu Tișa and collaborators (2024) in children aged 2–18 years from the north-western region of Romania, who reported the presence of a vitamin D deficiency in 27% of the cases [29] and Herdea and collaborators (2024) who observed a prevalence of vitamin D deficiency of 36.5% in children aged between 2 and 17 years from Bucharest, Romania [32]. Xi et al. (2024) reported in a study conducted on children from Central China that the prevalence of vitamin D deficiency and insufficiency was 17.7% and 23.4%, respectively [33]. High values of vitamin D deficiency were also reported in Greece among children aged 1–18 years, where a prevalence of vitamin D deficiency of 27.1% was recorded [34]. A higher prevalence of vitamin D deficiency was reported among Afghan primary school children, with 85.5% showing deficiency and 52.4% experiencing severe deficiency [35].

The prevalence of vitamin D insufficiency, deficiency, and severe deficiency varied by age. The highest level of vitamin D insufficiency was observed in the 13–18 years age group (52.56%). Vitamin D deficiency and severe deficiency were higher in the 7–12 years age group compared to other age groups, with 31.74% of the study participants presenting with vitamin D deficiency and 5.74% having severe deficiency of this vitamin. A possible explanation is that, due to the heavy school curriculum, students have very little time for outdoor activities, which has led to a significant reduction in sun exposure. This has contributed to a higher prevalence of vitamin D deficiency in the 7–12 years and 13–18 years age groups, compared to the 1–6 years age group. Considering that playing outdoors for at least 15 min a day with arms and legs uncovered between 10:00 a.m. and 3:00 p.m. from May to October has been shown to be sufficient to ensure an adequate level of vitamin D throughout the winter for children with fair skin [36], it is essential to increase the time spent outdoors during childhood by promoting outdoor play in schools throughout all seasons [37].

Following the evaluation of vitamin D status based on residence area, children living in urban areas showed a higher prevalence of vitamin D insufficiency (34.38%), deficiency (15.51%), and severe deficiency (1.99%), compared to children from rural areas. Similar results were obtained by Manios and collaborators (2017) [38]. Children and adolescents in urban areas spend most of their free time engaged in indoor activities, such as using mobile phones, watching TV programs, or playing video games, to the detriment of outdoor activities, which may explain the increased prevalence of vitamin D deficiency among them [38,39]. Another possible explanation for children from rural areas preventing higher vitamin D levels than those from urban regions is their greater consumption of dairy products, fish, and eggs. A study by Avagyan et al. (2016) found that children in rural Nepal may have higher vitamin D levels due to their diet, which includes these nutrient-rich foods [27].

Cases of vitamin D insufficiency, deficiency, and severe deficiency were more frequent among female subjects, who showed higher values of vitamin D insufficiency (34.85% vs. 33.63%), as well as deficiency (16.01% vs. 13.21%) and severe deficiency (2.22% vs. 1.58%) compared to male subjects. The results of this study are consistent with other studies conducted in Romania on children aged 0–17 years [40] and 2–18 years [29]. The presence of a higher incidence of vitamin D deficiency in female subjects compared to male subjects was also reported in other studies conducted in Saudi Arabia on children aged 0–21 years [7], in India among children aged 1–18 years [21], in Kuwait among children aged 11–16 years [41] and in Sri Lanka among children aged 10–18 years [42].

The highest values of vitamin D deficiency among the children included in this study were recorded in March (25.14%), which is consistent with previous studies conducted on the general population in Romania by Chirita-Emandi and collaborators (2015) [30] and Niculescu and collaborators (2017) [43], as well as with research conducted in Ukraine by Grygorieva and collaborators (2023) [44]. The highest level of vitamin D was recorded in September (69.92%), similar to the data reported in Romania by Niculescu and collaborators (2017) for the general population [43].

Vitamin D levels were lower in the winter months compared to the summer months. These results are consistent with the study conducted by Van de Walle and collaborators (2024) in Belgium on children aged 0–18 years, which reported higher vitamin D values in the summer/fall season compared to the winter and spring months [37]. This significant seasonal variation is in accordance with other studies that have demonstrated that seasonal fluctuations in sun exposure affect vitamin D levels [45,46]. During the winter period, the reduced number of sunlight hours and limited sun exposure led to a significant decrease in exposure to UVB radiation, which is essential for the endogenous synthesis of vitamin D [46]. The low level of 25(OH)D during the winter was correlated with a decrease in UVB radiation [47,48]. Therefore, to maintain their vitamin D levels, people primarily rely on dietary sources and on the vitamin D reserves accumulated during the summer through sun exposure [46].

Vitamin D deficiency screening can contribute to improving the health of children with low vitamin D levels by early identification of this deficiency, before the onset of adverse effects [49]. In clinical practice, there are numerous controversies regarding testing and administering vitamin D supplements, which highlights the need for the development of guidelines to improve the effectiveness of treatment [50,51].

Continuous monitoring of vitamin D levels in the pediatric population is essential for the development of effective public health programs and policies, such as the development of new strategies for vitamin D supplementation or food fortification. Food fortification with vitamin D is an effective strategy for increasing vitamin D intake among populations with limited sun exposure or inadequate dietary intake [46]. Fortified foods should be consumed regularly by the majority of the population [52].

To improve vitamin D status in the general population, it is important to implement public health strategies that take into account both individual and regional variables, such as latitude, genetic factors, lifestyle, body composition, and dietary intake [53,54,55,56,57].

Given the lack of information regarding vitamin D deficiency among children aged 1 to 18 years in Western Romania, this study provides relevant data on the prevalence of this deficiency in this region, suggesting the need for the implementation of appropriate measures to prevent this deficiency, such as the introduction of national prophylactic vitamin D supplementation or food fortification policies in children. It is important to mention that Nordic Countries such as Sweden have successfully implemented mandatory programs for food fortification and in Denmark, Finland, Iceland and Norway, voluntary food fortification policies [58]. The American Academy of Pediatrics recommended a minimum daily intake of 400 international units (IU) of vitamin D for all children, and adolescents [59]. However, evidence from an Iranian study on adolescent girls suggested that 800 IU or 1000 IU per day is insufficient to maintain serum 25-hydroxyvitamin D above 20 ng/mL [60]. Also, Kumaratne et al. [61] found that supplementation with 2000 IU daily for three months normalized vitamin D status in 80.39% (41/309) of adolescent participants from the USA who were otherwise healthy but deficient in vitamin D. Further research should focus on longitudinal studies in deficient children from Western Romania to assess the efficacy and adequate dosage of vitamin D supplementation especially in adolescents and urban residents.

This study revealed a higher degree of adequate vitamin D during the COVID-19 pandemic (2020) compared to 2018. Vitamin D was reported to support immunity and may reduce COVID-19 severity in children by regulating inflammation and respiratory health [62]. Our results are in contrast with Beyazgul and collaborators (2022) who reported lower levels of Vitamin D in children from Turkey after the pandemic period [63]. A possible explanation is that due to increased emphasis on vitamin D supplementation in children during the COVID-19 pandemic, more families from Western Romania may have prioritized supplementation as a preventive measure, as reported in other regions of the world [64].

Our work presents the following strengths: (I.) is the first study in over a decade to provide data on vitamin D levels in children from Western Romania, a region with distinct climatic and habitual characteristics and, therefore, underscoring the importance of localized screenings and interventions for international readers; (II.) while grounded in a specific region, the study contributes to global discourse by presenting data from an underrepresented geographic area; and (III.) it included non-hospitalized participants with no major diseases who came for a routine blood draw.

We acknowledge the following limitations: (I.) A notably higher number of children aged between 1 and 6 years old were included when compared with the other age groups; (II.) also there were more urban residents when compared to rural residents; (III.) the nurses conducting the blood draw asked the participants or their primary caregiver about the presence of severe diseases, medication intake and no questionnaires to collect data on participants associated conditions, body mass index (BMI), intake of supplements, or dietary habits, time spent inside/outside; (IV.) we did not perform Bayesian analysis due to the absence of pre-existing data from the region, which could potentially offer more nuanced insights by integrating prior knowledge with observed data; (V.) due to their single-time-point nature, cross-sectional studies are limited in their ability to establish causation or sequence events and primarily serve to reveal associations [65]; and lastly (VI.) we did not analyse the participants’ Fitzpatrick skin types.

Our study offers a limited snapshot in time of the status of vitamin D in children from Western Romania. In order to obtain a comprehensive understanding, large-scale studies using specialized questionnaires should be conducted in this region

## 5. Conclusions

Our study found the highest rates of vitamin D insufficiency in adolescents (13–18 years), urban residents, and children evaluated during the winter months. Screening and educational programs should be directed toward high-risk populations, including older children and urban residents. Further research should focus on establishing the efficacy of vitamin D supplements and their optimal dosage as well as age-based recommendations in this specific region.

## Figures and Tables

**Table 1:** Ordinal univariate logistic regression analysis of vitamin D status in children from Western Romania across age groups, area of residence and gender.

Variable (Total)	Vitamin D Status				Ordinal Univariate Analysis		
Age Groups	Severe Deficiency (<12 ng/mL) (%)	Deficiency (12–20 ng/mL) (%)	Insufficiency (21–30 ng/mL) (%)	Sufficiency (>30 ng/mL) (%)	cOR	95% CI	*p*-Value
1–6 (893)	8 (0.9)	49 (5.49)	186 (20.83)	650 (72.79)		Ref.	
7–12 (356)	20 (5.62)	113 (31.74)	159 (44.66)	64 (17.98)	0.17	0.13–0.21	<0.001
13–18 (449)	4 (0.89)	85 (18.93%)	236 (52.56)	124 (27.62)	0.08	0.06–0.11	<0.001
Area of residence							
Rural (389)	6 (1.54)	44 (11.31)	131(33.68)	208 (53.47)		Ref.	
Urban (1309)	26 (1.99)	203 (15.51)	450 (34.38)	630 (48.13)	0.78	0.63–0.97	0.03
Gender							
Female (812)	18 (2.22)	130 (16.01)	283 (34.85)	381 (46.92)		Ref.	
Male (886)	14 (1.58)	117 (13.21)	298 (33.63)	457 (51.58)	1.23	1.02–1.47	0.03
Total (1698)	32 (1.88)	247 (14.55)	581 (34.22)	838 (49.35%)			

**Table 2:** Monthly variation in vitamin D status of children from Western Romania.

Variable (Total)	Vitamin D Status				Univariate Analysis		
Month	Severe Deficiency (<12 ng/mL) (%)	Deficiency (12–20 ng/mL) (%)	Insufficiency (21–30 ng/mL) (%)	Sufficiency (>30 ng/mL) (%)	cOR	95% CI	*p* -Value
January (147)	9 (6.12)	22 (14.97)	57 (38.78)	59 (40.14)		Ref.	
February (171)	3 (1.75)	30 (17.54)	68 (39.77)	70 (40.94)	1.09	0.72–1.65	0.67
March (179)	7 (3.91)	45 (25.14)	70 (39.11)	57 (31.84)	0.69	0.46–1.04	0.08
April (131)	2 (1.53)	28 (21.37)	51 (38.93)	50 (38.17)	0.95	0.61–1.47	0.81
May (182)	2 (1.1)	32 (17.58)	68 (37.36)	80 (43.96)	1.21	0.80–1.82	0.36
June (159)	3 (1.89)	29 (18.24)	45 (28.3)	82 (51.57)	1.47	0.96–2.26	0.08
July (134)	4 (2.99)	12 (8.96)	38 (28.36)	80 (59.7)	2.22	1.41–3.49	0.001
August (113)	0 (0)	5 (4.42)	33 (29.2)	75 (66.37)	3.21	1.97–5.23	<0.001
September (123)	0 (0)	8 (6.5)	29 (23.58)	86 (69.92)	3.61	2.22–5.88	<0.001
October (126)	1 (0.79)	10 (7.94)	33 (26.19)	82 (65.08)	2.87	1.79–4.59	<0.001
November (126)	0 (0)	13 (10.32)	55 (43.65)	58 (46.03)	1.5	0.96–2.33	0.08
December (107)	1 (0.93)	13 (12.15)	34 (31.78)	59 (55.14)	1.88	1.17–3.03	0.01
Total (1698)	32 (1.88%)	247 (14.55)	581 (34.22)	838 (49.35)			

**Table 3:** Ordinal univariate logistic regression of annual trends in vitamin D status in children from Western Romania.

Variable (Total)	Vitamin D Status				Ordinal Univariate Analysis		
Year	Severe Deficiency (<12 ng/mL) (%)	Deficiency (12–20 ng/mL) (%)	Insufficiency (21–30 ng/mL) (%)	Sufficiency (>30 ng/mL) (%)	cOR	95% CI	*p*-Value
2018 (369)	4 (1.08)	61 (16.53)	131 (35.5)	173 (46.88)		Ref.	
2019 (528)	9 (1.7)	70 (13.26)	179 (33.9)	270 (51.14)	1.19	0.92–1.52	0.19
2020 (473)	11 (2.33)	51 (10.78)	156 (32.98)	255 (53.91)	1.33	1.03–1.72	0.03
2021 (328)	8 (2.44)	65 (19.82)	115 (35.06)	140 (42.68)	0.81	0.61–1.07	0.13
Total (1698)	32 (1.88)	247 (14.55)	581 (34.22)	838 (49.35)			

**Table 4:** Multivariate analysis of vitamin D status in children from Western Romania.

	Multivariate Analysis		
Variable	aOR	95% CI	*p*-Value
Age groups			
7–12	0.16	0.13–0.21	<0.001
13–18	0.07	0.05–0.09	<0.001
Year			
2019	1.49	1.14–1.97	0.004
2020	1.73	1.29–2.32	<0.001
2021	1.63	1.19–2.24	0.003
Month			
July	2.61	1.58–4.30	<0.001
August	3.71	2.16–6.38	<0.001
September	5.47	3.17–9.42	<0.001
October	4.52	2.68–7.64	<0.001
November	1.83	1.13–2.96	0.013
December	2.2	1.31–3.71	0.003

## Data Availability

All relevant data are presented in the study.
